# Weighted gene co-expression network analysis and CIBERSORT screening of key genes related to m6A methylation in Hirschsprung’s disease

**DOI:** 10.3389/fgene.2023.1183467

**Published:** 2023-04-18

**Authors:** Jiaqian Huang, Tingwei Chen, Junjie Wang, Zhiqiang Wang, Shungen Huang

**Affiliations:** ^1^ Pediatric Surgery, Children’s Hospital of Soochow University, Suzhou, China; ^2^ Department of Biochemistry and Molecular Biology, Medical College, Soochow University, Suzhou, China

**Keywords:** hirschsprung’s disease, M6A, WGCNA, CIBERSORT, enrichment analysis

## Abstract

Hirschsprung’s disease (HSCR) is a neural crest disease that results from the failure of enteric neural crest cells (ENCCs) to migrate to the corresponding intestinal segment. The RET gene, which regulates enteric neural crest cell proliferation and migration, is considered one of the main risk factors for HSCR and is commonly used to construct HSCR mouse models. The epigenetic mechanism of m6A modification is involved in HSCR. In this study, we analyzed the GEO database (GSE103070) for differentially expressed genes (DEGs) and focused on m6A–related genes. Comparing the RNA-seq data of Wide Type and RET Null, a total of 326 DEGs were identified, of which 245 genes were associated with m6A. According to the CIBERSORT analysis, the proportion of Memory B-cell in RET Null was significantly higher than that of Wide Type. Venn diagram analysis was used to identify key genes in the selected memory B-cell modules and DEGs associated with m6A. Enrichment analysis showed that seven genes were mainly involved in focal adhesion, HIV infection, actin cytoskeleton organization and regulation of binding. These findings could provide a theoretical basis for molecular mechanism studies of HSCR.

## 1 Introduction

HSCR is a common digestive malformation in children, with a prevalence of 1/5000 to 1/2000, ranking second in digestive malformations (Gao et al., 2022). It is more prevalent in males, with an average male-to-female ratio of 4:1 (Pakarinen, 2018). The main clinical manifestations of HSCR include absent feces or delayed fetal excretion, bilious vomiting, feeding difficulties, and intractable constipation. Hirschsprung-associated enterocolitis (HAEC) occurs in some children, with a prevalence of 18.5% preoperatively and 18.2% postoperatively (Hagens et al., 2022). The symptoms include abdominal distention, fever, diarrhea, vomiting, bloody stools, and constipation (Gosain et al., 2017). However, some children exhibit atypical results in preoperative barium enema and rectal-anal manometry tests. Additionally, intestinal tissue damage with bleeding and perforation may occur after biopsy. The underlying cause of HSCR is the absence of ganglion cells in the intermuscular plexus of the intestinal wall due to arrested development of ENCCs migration. Mutations in genes, such as RET, EDNRB, and SOX10 have been associated with the pathogenesis of HSCR(Sergi et al., 2017). Among them, RET is the major risk factor for HSCR, which is involved in the proliferation, migration, and differentiation of intestinal neural crest cells (Okamoto et al., 2021). The tyrosine kinase receptor encoded by the RET proto-oncogene plays a critical role in the development of the enteric nervous system. Aberrant expression of the RET gene can result in abnormal colonization of ENCCs within the intestine and even promote ENCCs apoptosis, ultimately leading to the occurrence of HSCR. RET knockout is a conventional method to build the model of HSCR in mice (Soret et al., 2020).

N6-methyladenosine (m6A) methylation is a common epigenetic modification in eukaryotic, accounting for 80% of RNA methylation modifications, and it exerts biological functions by affecting RNA metabolism (Jiang et al., 2021). The formation of m6A is catalyzed by a methyltransferase complex (MTC), which comprises genes such as METTL3, METTL14, METTL16, and WTAP. The m6A demethylase, also known as the “eraser,” is involved in the removal of m6A, while RNA reader proteins recognize and bind to m6A, thus achieving corresponding functions. Related genes involved in this process include YTHSCRC2, YTHSCRF1, LRPPRC, HNRNPA2B1, and IGFBP1(Zaccara et al., 2019). Methyltransferases can catalyze the m6A modification of adenosine on mRNA, while the role of demethylases is to demethylate the bases that have undergone m6A modification. The function of reader proteins is to recognize the bases that have undergone m6A modification, thus activating downstream regulatory pathways, such as RNA degradation and miRNA processing. The m6A demethylase, ALKBH5, has been found to inhibit the proliferation and migration of enteric neural crest cells by upregulating TAGLN and is involved in the pathogenesis of HSCR(Wang B. et al., 2021).

B lymphocytes (B-cell) are a complex cell type that plays a central role in humoral immunity against infection, autoimmunity and transplantation. B-cell activation leads to the formation of memory B-cell populations (Frasca et al., 2020). Memory B-cell can be classified based on the immunoglobulin markers they express, including IgM^+^, IgG^+^, IgA^+^, and IgE^+^ memory B-cell. Tissue-resident memory B-cell have been reported to be predominantly present in the intestine and characterized by the expression of CD45RB and CD69 (Weisel et al., 2020), providing the first line of defense against reinfection. IgM^+^ memory B-cell that are distributed throughout the intestine are closely related to IgM^+^ plasma cells in the gut and play a unique role in mucosal protection. B-cell can impede the interaction between epithelial and stromal cells and play a negative role in repairing intestinal damage (Frede et al., 2022).

In this study, we obtained RNA-seq data of Wide Type and RET Null mouse intestinal samples from the GEO database (GSE103070), and analyzed the DEGs associated with m6A modification. We used weighted gene co-expression analysis and the CIBERSORT algorithm to screen for differential genes associated with m6A methylation modifications in memory B-cell of RET Null mouse samples. Enrichment analysis was performed to develop a theoretical framework for the diagnosis and study of HSCR.

## 2 Materials and methods

### 2.1 Data collection and processing

The Gene Expression Omnibus (GEO) database (https://www.ncbi.nlm.nih.gov/) provides publicly available data. We identified the GSE103070 dataset by searching the GEO database using keywords such as RET and Gut. The contributors of the GSE103070 dataset have confirmed significant changes in gene expression during late-stage development of the intestine (Chatterjee et al., 2019). By analyzing data from three time points, E10.5, E12.5, and E14.5, we confirmed that there were substantial differences in gene expression at E14.5. Therefore, we focused our analysis and discussion on the data from E14.5.

### 2.2 Differential expression gene analysis

In R (4.2.0) software, data from the above RNA-seq were analyzed using the R package “limma” to identify differentially expressed genes (DEGs). Significantly DEGs were detected according to the following criteria: (1) |log_2_FC| > 0.5, (2) False discovery rate (FDR) adjusted *p*-value <0.05. The “ggplots” and “pheatmap” packages in R were utilized to create volcano plots and heatmaps depicting the DEGs.

### 2.3 Identification of m6A-related genes

Pearson correlation analysis was conducted to evaluate the association between m6A modifications and DEGs in Wide Type and RET Null mouse intestinal tissues, with a threshold of |Pearson R|>0.5 and *p* < 0.05.

### 2.4 Immune cell abundance analysis

CIBERSORT is a general deconvolution algorithm based on gene expression, which can estimate the relative proportion of 22 types of immune cells (Le et al., 2021), such as M1 macrophages, M2 macrophages, memory B-cell and mast cells from gene expression profiles. The relative abundance of the 22 immune cell types in the Wide Type and RET Null samples was assessed by CIBERSORT.

### 2.5 WGCNA analysis

To explore the set of modular genes associated with the immune cells in RET Null sample, genes with mean FPKM>5 were selected for sample clustering. The soft threshold for subsequent network construction was calculated by pickSoftThreshold in the WGCNA package based on the correlation coefficient between the β value and the mean gene connectivity. The topological overlap matrix (TOM) was constructed using BlockwiseModules, and clustering was performed using the dynamic tree shearing algorithm. Each co-expression module was assigned a unique color to distinguish genes with similar expression patterns.

### 2.6 Venn diagram and enrichment analysis

The overlap between immune cell-related genes and m6A-related DEGs was examined using a Venn diagram generated with the online tool (https://bioinfogp.cnb.csic.es/tools/venny/). For enrichment analysis of genes in the identified modules and to explore their biological functions, Metascape (https://metascape.org/gp/index.html) was employed, which offers extensive gene function annotation and pathway analysis capabilities.

## 3 Results

### 3.1 Screening for differentially expressed genes

**FIGURE 1:**
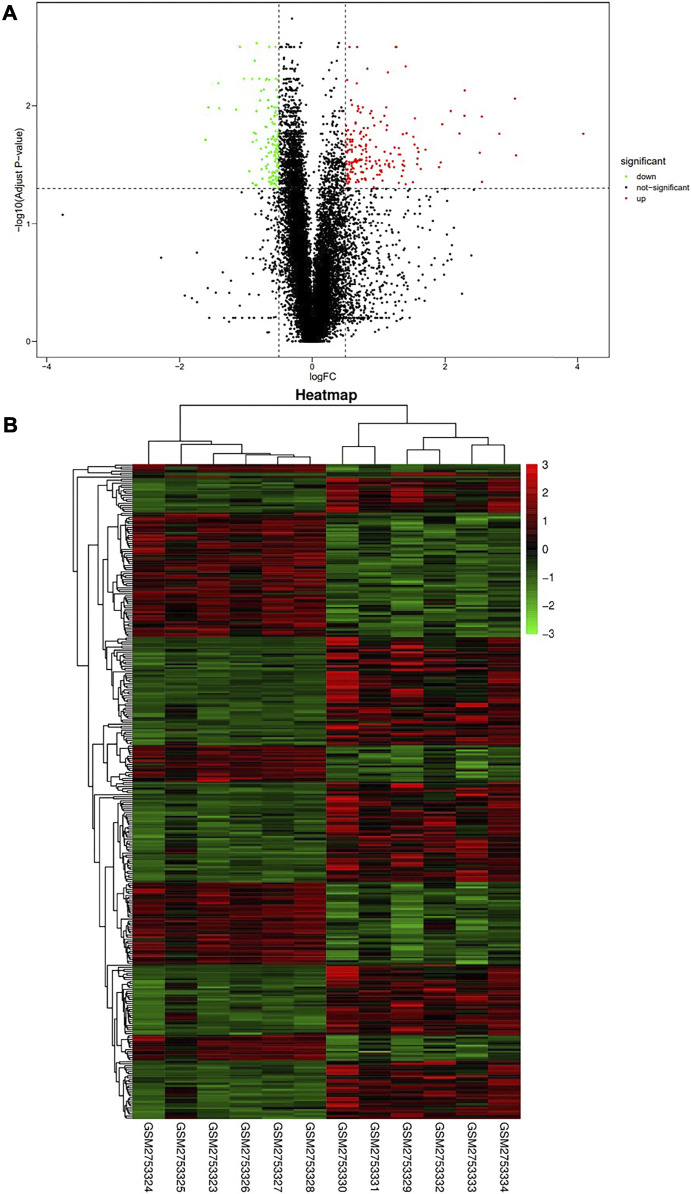
Screening of differentially expressed genes in Wide Type and RET Null samples. **(A)** Volcano plot of differentially expressed genes (red dots represent upregulated genes, green dots represent downregulated genes); **(B)** Heat map of differentially expressed genes (high gene expression levels were indicated in red, low gene expression levels were indicated in green). GSM2753324-28 were RET Null and GSM2753329-34 were Wide Type.

We identified 326 genes (Supplementary Material S1) as differentially expressed genes by screening the RNA-seq data of E14.5 in the GSE103070 dataset with |log_2_FC| >0.5 and adjusted *p* < 0.05, including 139 upregulated genes and 187 downregulated genes. A volcano map was created using DEG analysis results, with upregulated genes shown in red and downregulated genes shown in green (Figure 1A). The heat map was also plotted, with each column of the heat map representing a sample and each row representing a gene (Figure 1B). Similar genes and samples were clustered in the horizontal and vertical coordinates. We were able to observe that the expression patterns of functionally related genes were also similar.

### 3.2 Analysis of differentially expressed genes associated with m6A

**FIGURE 2:**
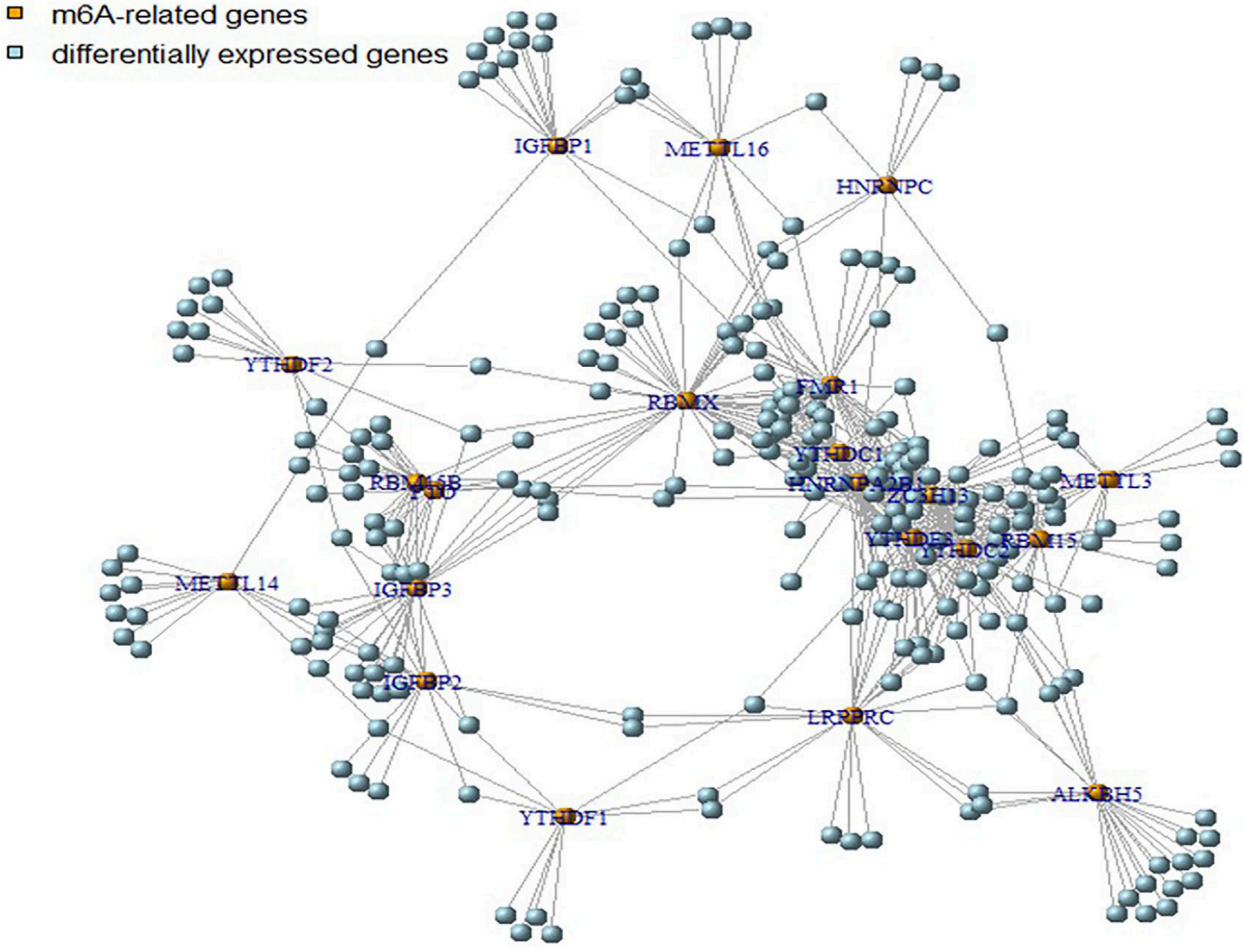
Screening for m6A-related DEGs. Red dots represent genes involved in m6A modification, blue dots represent DEGs, and lines represent the correlation between dots.

Using Pearson correlation analysis, we identified 245 genes associated with m6A regulators by applying the screening conditions of |Pearson R|>0.5 and *p* < 0.05. M6A-modified genes were depicted with red dots in the figure, and differentially expressed genes were shown with blue dots (Figure 2). In summary, 245 genes were aberrantly expressed in HSCR and may be related to m6A methylation modification.

### 3.3 Assessment of immune cell abundance

**FIGURE 3:**
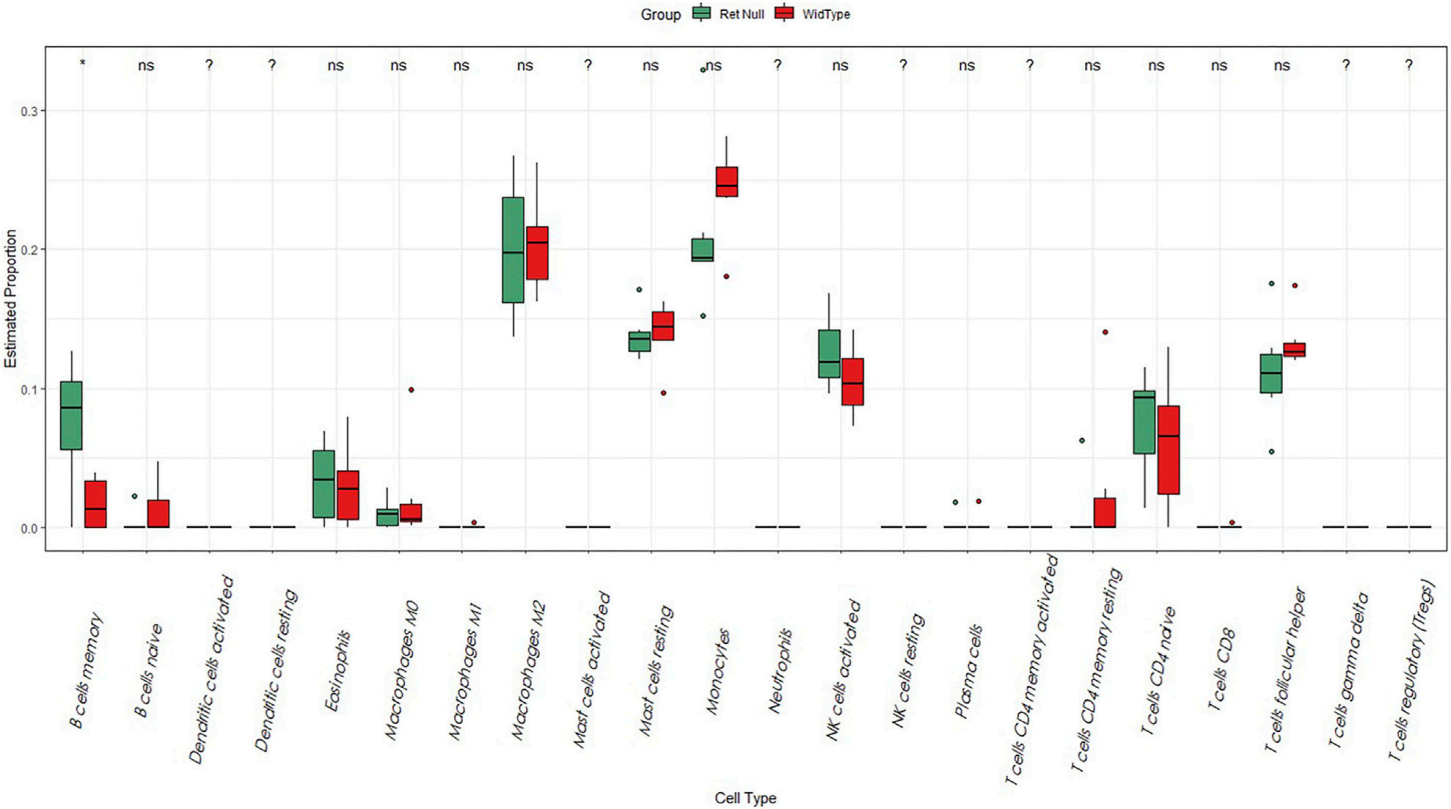
Box plot of the proportion of immune cells in Wide Type and RET Null mouse intestinal samples. The horizontal coordinate is the type of immune cells and the vertical coordinate is the proportion of immune cells. Green represents the proportion of immune cells in RET Null mouse intestinal samples, red represents the proportion of immune cells in Wide Type, and *p* < 0.05 is marked as “*”.

In our study, we analyzed the abundance of immune cells in both Wide Type and RET Null samples using the CIBERSORT algorithm. R language was used to visualize the distribution of 22 immune cell types, with red representing Wide Type and green representing RET Null (Figure 3). Our analysis revealed a significant difference in the proportion of memory B-cell between RET Null and control samples, suggesting that memory B-cell may play an important role in the development of HSCR.

### 3.4 Construction and screening of WGCNA modules

**FIGURE 4:**
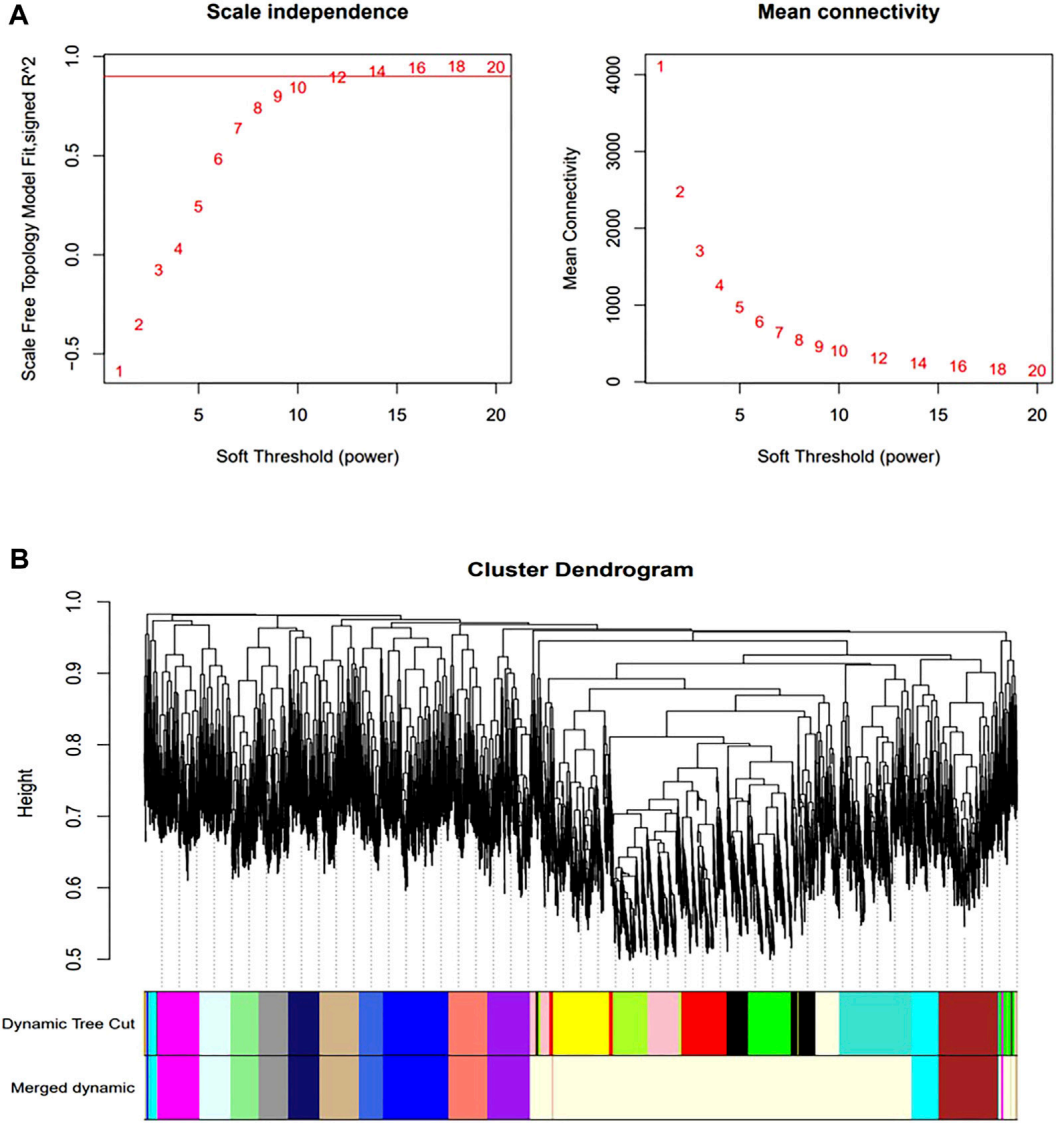
Construction of co-expression network. **(A)** Determination of optimal soft threshold (during module selection, the adjacency matrix was transformed into a topological matrix to determine the optimal soft threshold β = 12); **(B)** Clustering tree of co-expressed gene modules (similar genes were grouped into the same module by dynamic splicing and cluster analysis).

**FIGURE 5:**
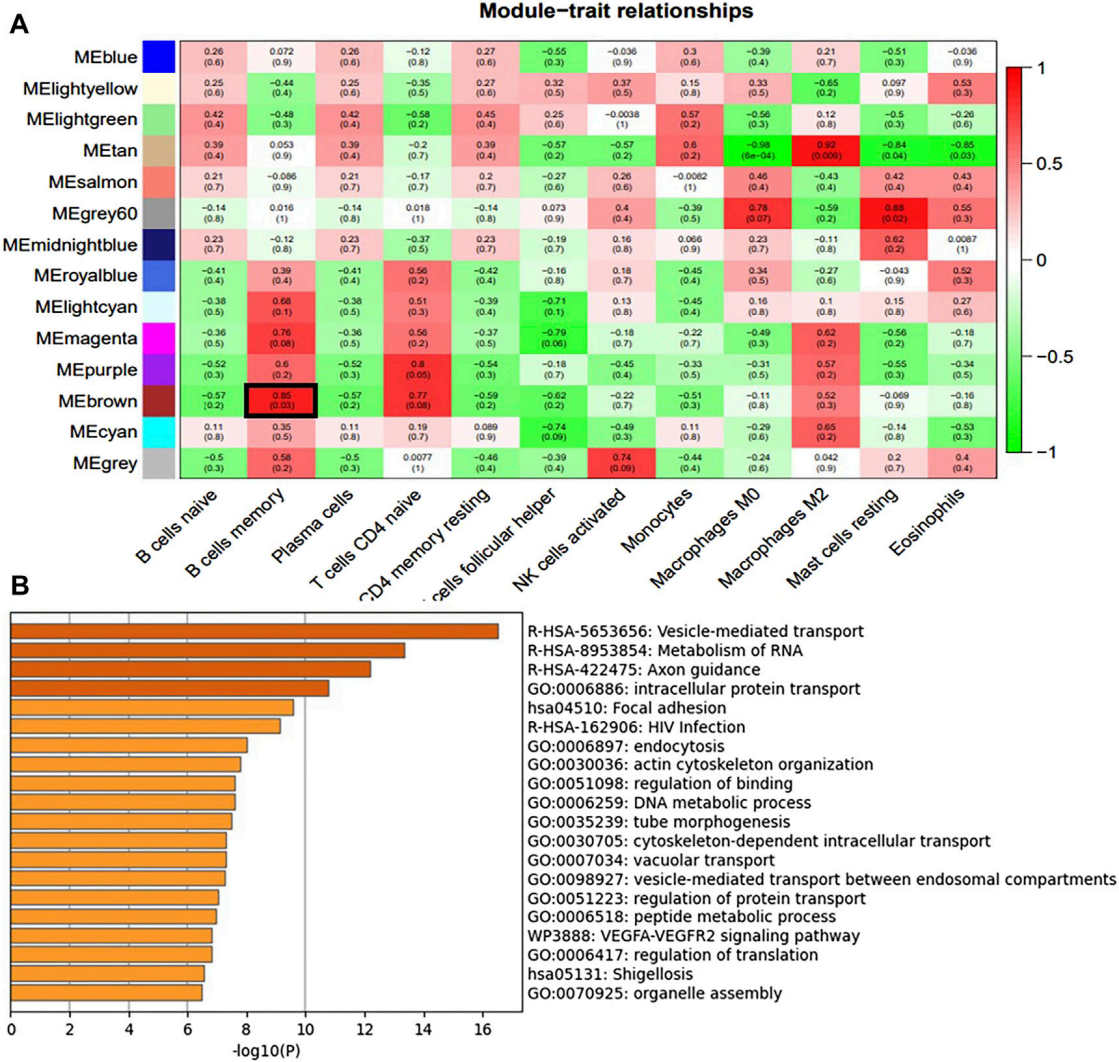
Immune cell module and Enrichment Analysis. **(A)** Correlation between module genes and immune cells (the redder the color, the higher the correlation; Pearson correlation coefficient between module characteristic genes and sample characteristic vectors, and the number in brackets represents the corresponding *p*-value) **(B)** Enrichment analysis of brown module genes most associated with memory B-cell.

We constructed a gene co-expression network using WGCNA to identify modules associated with HSCR samples with the highest correlation with immune cells. The scale-free topological fit index R2 was 0.9 and βwas 12 (Figure 4A). After identifying the gene modules using the dynamic tree-cutting method, we calculated the feature vector values for each module. Modules with similar distances were merged (Figure 4B). Different types of immune cells were represented by the horizontal coordinates, and different module genes were represented by the vertical coordinates. Darker colors represented higher correlations. Red represents positive correlations and green represents negative correlations. Since there is a significant difference in the proportion of memory B-cell between RET Null mouse intestinal samples and Wide Type samples, subsequent studies will focus on the brown module of memory B-cell genes (Figure 5A).

### 3.5 Identification of key genes

**FIGURE 6:**
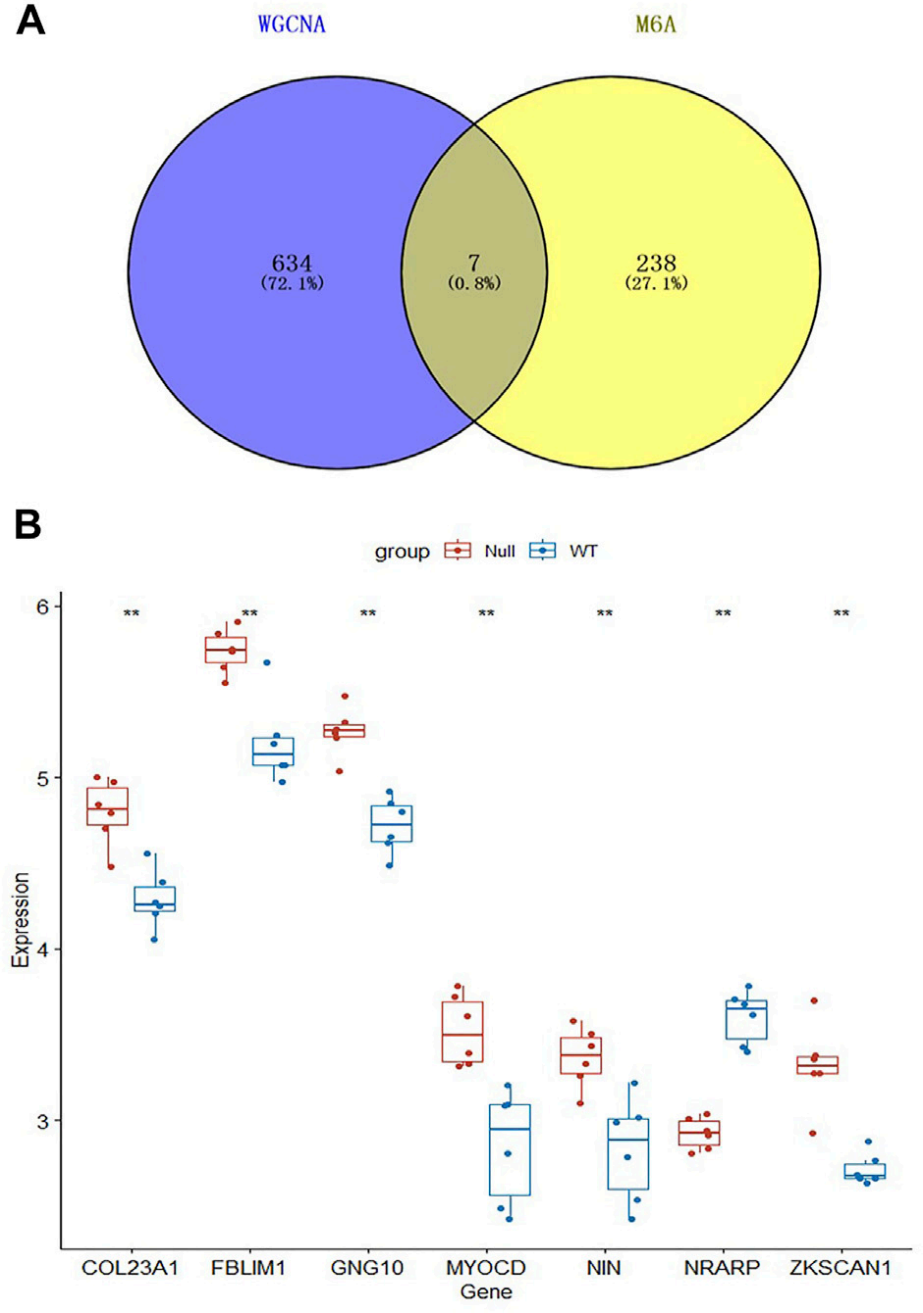
Screening and expression analysis of key genes. **(A)** Intersection of m6A-related DEGs with brown module genes. **(B)** Expression box plot of the seven key genes, Null represents RET knockout and WT represents Wide type.

To identify differentially expressed genes associated with memory B-cell and related with m6A modifications, we mapped 245 DEGs associated with m6A and 641 genes associated with the most relevant modules of memory B-cell in a Venn diagram (Figure 6A), resulting in seven (COL23A1, FBLIM1, GNG10, MYOCD, NIN, NRARP, and ZKSCAN1) differentially expressed genes. COL23A1, FBLIM1, GNG10, MYOCD, NIN and ZKSCAN1 were highly expressed in the RET Null group, while NRARP was low expressed (Figure 6B). Enrichment analysis of the brown module genes (Figure 5B) revealed that these seven genes were mainly involved in biological processes, such as local adhesion, HIV infection, actin cytoskeleton organization, binding regulation, and microtubule morphogenesis.

## 4 Discussion

HSCR is a common congenital gastrointestinal disorder in children, characterized by a congenital absence of intrinsic ganglion cells in the muscular and submucosa plexuses of the gastrointestinal tract (Heuckeroth, 2018). The mechanism of HSCR is the disturbance of the proliferation and migration of ENCCs during embryonic development or alteration of the intestinal microenvironment (Nagy et al., 2021). Genetic studies have found that genes, such as RET and EDNRB are associated with HSCR(Bachetti and Ceccherini, 2020). RET is a major risk factor for HSCR that encodes tyrosine kinase and is closely associated with the proliferation, migration, and differentiation of ENCCs. M6A modification is involved in the occurrence and progression of HSCR, and this study focuses on the possible role of m6A modification-related genes in HSCR after RET knockdown.

In this study, we analyzed the GSE103070 dataset from the GEO database using the CIBERSORT algorithm. Our results showed that the proportion of memory B-cell was significantly increased in intestinal samples from RET Null compared to the Wide Type. Using WGCNA, we identified gene modules with the highest correlation with immune cells and intersected them with m6A-related differentially expressed genes to identify seven key genes (COL23A1, FBLIM1, GNG10, MYOCD, NIN, NRARP and ZKSCAN1) significantly associated with m6A and memory B-cell.

Studies have reported that the expression of memory B-cell is significantly different in patients with ulcerative colitis compared to normal subjects (Pararasa et al., 2019). In hepatoblastoma, the relative abundance of memory B-cell is positively correlated with disease severity (Liu et al., 2022). In HSCR, abnormalities in gut microbial regulation usually cause HAEC, resulting in delayed thymic development. This study found that memory B-cell were more predominant in the RET Null HSCR model, suggesting their close association with HSCR. Therefore, we performed a follow-up analysis of memory B-cell.

Enrichment analysis of memory B cell-associated genes revealed that the seven candidate genes are mainly involved in several biological processes, including focal adhesion, HIV infection, actin cytoskeleton organization, regulation of binding, tube morphogenesis, and the VEGFA-VEGFR2 signaling pathway. By checking through the RMDisease2 website, we found that six of the key genes can be modified by m6A. In multiple sclerosis (MS), CD38^+^ memory B-cell in the blood migrate toward the central nervous system by expressing the adhesion molecule ALCAM(McWilliam et al., 2018). The downregulation of p130Cas in ENCCs in the presence of collagen ColVI affects FN-induced actin polymerization and focal adhesion formation (Nishida et al., 2018). Notch-regulated ankyrin repeat-containing protein (NRARP) is a small protein with two anchor protein repeat sequences that can regulate neural crest cell differentiation by regulating LEF1 stability (Ishitani et al., 2005). NRARP protein is required for the anterior-posterior somite pattern in mice, and the reduced expression of constitutive NRARP blocks early thymic cell maturation progression (Bertout et al., 2009). In our findings, NRARP expression was significantly reduced in intestinal samples from RET Null mice, suggesting that the neural crest cell differentiation involving NRARP is closely related to HSCR. Serine-binding LIM protein 1 (FBLIM1) is involved in cell attachment and its encoded Migfilin is essential for cellular processes, such as cell morphology and migration. FBLIM1 can co-localize with kindlin-1 and kindlin-2 on the adhesion spots of normal human keratin-forming cells (Brahme et al., 2013). The regulation of Migfilin expression is essential for neuronal development (Ishizuka et al., 2018). G protein-coupled membrane receptors regulate intercellular transmitters and hormone secretion in neuroendocrine cells. GNG10 encodes a subunit of G proteins involved in cell cycle regulation and is closely associated with the intestinal microflora. Overexpression of GNG10 promotes colorectal cancer progression (Wang N. et al., 2021), and we found that GNG10 expression was higher in the RET Null, suggesting an association between GNG10 and HSCR. ZKSCAN1 acts as a cell cycle regulator to regulate cell progression (Zhu et al., 2019). MYOCD can inhibit the developmental program through the NOTCH signaling pathway, transforming vascular smooth muscle cells (VSMC) into a dedifferentiated state, leading to complete transformation into macrophage-like cells (Zhang et al., 2022). The COL23A1 gene can be used as a surrogate marker for monitoring disease activity in ulcerative colitis and Crohn’s disease (Manon-Jensen et al., 2021), as well as a biomarker for papillary thyroid cancer (Pan et al., 2021) and clear cell renal cell carcinoma (Xu et al., 2017).

Our findings suggest that the key genes associated with memory B-cell and m6A modification may influence the development of HSCR by participating in the inflammatory response and affecting neural crest cell proliferation, migration, and differentiation. However, due to limited data availability on human samples in HSCR, further exploration is required. We will conduct clinical validation of the genes identified in this study to determine their potential as new therapeutic targets, providing a theoretical basis for clinical diagnosis and a deeper understanding of HSCR mechanisms.

## Data Availability

The datasets presented in this study can be found in online repositories. The names of the repository/repositories and accession number(s) can be found in the article/Supplementary Material.
